# Lower hepatitis E clearance rates in thoracic organ recipients compared to visceral organ recipients: an effect of stronger immunosuppression?

**DOI:** 10.1007/s15010-026-02892-x

**Published:** 2026-08-11

**Authors:** Sven Pischke, L. Kwoka, A. Nolde, T. B. Huber, M. Sterneck, F. Berisha, T. Fründt, L. Fischer, M. J. Barten, S. Blankenberg, M. Kluger, M. Mahmud, M. Rybczynski, S. Huber, A. Lohse, H. Reichenspurner, B. Sill, A. Bernhardt, F. Grahammer, H. F. E. Klose, J. Schulze zur Wiesch, S. Polywka

**Affiliations:** 1 I. Department of Medicine, University Medical Center Hamburg-Eppendorf, Hamburg, Germany; 2 German Center for Infection Research (DZIF), Hamburg-Lübeck-Borstel-Riems Partner Site, Hamburg, Germany; 3 Abteilung für Pneumologie, II. Medizinische Klinik und Poliklinik, Universitätsklinikum Hamburg-Eppendorf, Hamburg, Deutschland; 4 III. Department of Medicine, University Medical Center Hamburg-Eppendorf, Hamburg, Germany; 5 Department of General and Interventional Cardiology, University Heart and Vascular Center Hamburg, Hamburg, Germany; 6 Department of Visceral Transplantation, University Medical Center Hamburg-Eppendorf, Hamburg, Germany; 7 Department of Cardiovascular Surgery, University Medical Center Hamburg-Eppendorf, Hamburg, Germany; 8 Microbiology, University Hospital Hamburg-Eppendorf, Hamburg, Germany

**Keywords:** Hepatitis E, HEV, Transplant, Chronic Infection, Immunosuppression

## Abstract

**Background and aims:**

Hepatitis E virus (HEV) infection is a significant concern for solid organ transplant (SOT) recipients. However, the extent of this disease among recipients of different organs remains unclear.

**Methods:**

This retrospective cohort study reviewed 63 adult SOT patients with PCR-confirmed HEV infection, including liver, kidney, heart, and lung transplant recipients.

**Results:**

Chronic HEV infection (viral persistence beyond three months) was observed in 64% of infected patients, with a significantly higher occurrence in thoracic organ recipients compared to abdominal organ recipients. Ribavirin therapy, started in 40 patients with chronic infection, resulted in a sustained virological response in 78%. However, thoracic organ recipients exhibited notably lower clearance rates (55%) than abdominal organ recipients (91%). The use of tacrolimus, cyclosporine A, mycophenolate mofetil, azathioprine, or steroids was not linked to the development or eventual clearance of chronic infection. Nonetheless, 4 out of 25 patients (16%) receiving everolimus cleared the infection. spontaneously, compared to 20 of 39 patients (51%) on regimens without everolimus (*p* = 0.007). Low-dose maintenance ribavirin therapy proved effective in stabilizing liver function and, in some cases, achieving viral clearance. Despite treatment, four patients with chronic HEV infection died from cirrhosis.

**Conclusion:**

This study emphasizes the need for customized treatment Strategies for HEV infection in SOT recipients, highlighting the limitations of serology, the variable response depending on transplant type, and the potential role of long-term ribavirin therapy.

## Introduction

Hepatitis E is a disease whose importance in the Western world has changed significantly over the past 20 years [1–3]. This infection, caused by the hepatitis E virus (HEV, also known as *Paslahepevirus balayani*), was first identified in the 1980s and was traditionally viewed as a disease limited to tropical regions [4]. However, since the early 2000s, it has become evident that HEV genotype 3 infections also happen endemically in Europe and North America. Notably, a large meta-analysis found considerable epidemiological variation in HEV prevalence worldwide: estimated HEV PCR positivity rates range from 0.01% to 0.14%, with notably higher rates reported in Asia (0.14%) and Europe (0.10%) compared to North America (0.01%) [5].

In 2008, hepatitis E drew more attention when cases of chronic infection were reported in liver and kidney transplant recipients [2, 6–9]. Later, chronic HEV infection was also identified in heart and lung transplant recipients [10, 11]. The initial successful trials of antiviral treatment for chronic hepatitis E used interferon, either alone or in combination with ribavirin [12–14]. However, due to interferon’s notable side effects and its potential to cause organ rejection in transplant patients, ribavirin monotherapy was quickly considered an alternative. Early case reports and a small study of 10 patients showed that ribavirin could be safely given to transplant recipients with chronic hepatitis E and was associated with sustained virological response (SVR) in many cases [15, 16] ; however, in some cases, the treatment failed [16].

In 2014, a large multicenter cohort study involving 59 solid organ transplant recipients with chronic hepatitis E treated with ribavirin was published [17]. A sustained virological response (SVR) was achieved in 78% (*n* = 46) of patients, while 22% (*n* = 13) experienced virological relapse or breakthrough and were not cured. Among those 13 patients without initial SVR, 4 (31%) achieved viral clearance after a prolonged course of ribavirin re-treatment. Based on these findings, the authors recommended a three-month course of ribavirin for uncomplicated cases and an extended five-month regimen for patients who do not achieve SVR early [17].

In addition to the multicenter cohort study, a systematic review of 105 patients across 19 publications found that ribavirin monotherapy achieved a sustained virological response (SVR) in 78% of cases. However, because definitions of SVR varied across studies, only 64% of patients (67 out of 105) met a more stringent criterion—viral negativity for more than 6 months [18].

In addition to ribavirin monotherapy, achieving an SVR may also be possible by reducing immunosuppression, using interferon, or combining these approaches. However, it is crucial to remember that both interferon and lowering immunosuppression carry the risk of rejection and are, therefore, limited options, particularly for vulnerable patients such as those with heart and lung transplants. Furthermore, the direct-acting antiviral agent sofosbuvir has been linked to HEV clearance in some, but not all, isolated case reports [19–21]. However, a standardized pilot study involving 12 patients did not show any significant therapeutic effect [22]. Two mutations in the HEV genome—G1634R and A1343V—are linked to treatment failure during ribavirin or sofosbuvir therapy [23, 24]. An additional potentially relevant mutation, Y1320H, which causes ribavirin treatment failure, was identified in a rabbit model. However, these findings are based on genetic analyses of individual cases or experiments, and so far, no prospective randomized trials have assessed the impact of these mutations on treatment outcomes.

The goal of our current retrospective cohort study was to evaluate ribavirin use in solid-organ transplant recipients across different transplant types. By analyzing the progression of HEV infections in recipients of various organs, we aimed to provide evidence-based recommendations for other transplant centers regarding risk assessment and expected treatment outcomes.

## Patients and methods

This study was carried out at the University Medical Center Hamburg-Eppendorf, a leading tertiary care facility in northern Germany. All cases of PCR-confirmed HEV infection among adult solid organ transplant (SOT) recipients (*n* = 67) were retrospectively reviewed using the local Soarian electronic health record system.

In line with Kamar et al. and the EASL guidelines, chronic hepatitis E is defined as the presence of the virus for more than 3 months [25]. All patients in our study who were chronically HEV-infected met this criterion.

The following are our current guidelines for immunosuppression in patients who have undergone liver, kidney, heart, or lung transplants (as of April 2026). It should be noted that these guidelines have changed repeatedly over the past few decades.

For liver transplant recipients: Standard induction involves basiliximab plus steroids, followed by maintenance immunosuppression aimed at achieving a steroid-free regimen. The current protocol includes tacrolimus (target trough level: 4–5 ng/mL) plus mycophenolate mofetil 1000 mg/day. For specific cases such as tumor presence or renal insufficiency, a low dose of tacrolimus (target trough level: 3–4 ng/mL) combined with everolimus (3–4 ng/mL) is used.

For kidney transplant recipients: While ciclosporin A and azathioprine were standard therapies three decades ago, our current regimen involves induction with basiliximab and steroids. In high-risk groups, additional antithymocyte globulin is administered. During the first three months, tacrolimus is targeted at 8–10 ng/mL, and mycophenolate mofetil is given at 2000 mg or enteric-coated mycophenolate mofetil at 720 mg daily. Prednisone is administered for the duration of the transplant in cases of underlying immunological disease, re-transplantation, or other immunological risks at a dose of 5 mg. For all other patients, we aim to transition to a steroid-free regimen starting on day 8. From month four onward, the target tacrolimus trough level is 5–8 ng/mL, and in the long term, it is maintained at 5 ng/mL. Patients with previous nephrocellular carcinoma or significant CMV infections often receive everolimus. Those experiencing gastrointestinal side effects from mycophenolate mofetil typically switch to enteric-coated formulations.

For heart transplant recipients: A more robust triple immunosuppressive maintenance therapy includes tacrolimus (target trough levels: 8–10 ng/mL initially, or 10–12 ng/mL depending on immunological risk) and mycophenolate mofetil at 2000 mg/day or enteric-coated mycophenolate at 1440 mg/day. Dose reductions are usually made after six months, with further adjustments over time. Alternatively, cyclosporine (target trough level: 150–200 ng/mL) may be used in cases of tacrolimus intolerance or historically as standard therapy. Everolimus is prescribed based on our kidney transplant guidelines—especially for viral infections, malignancies, or adverse effects, particularly in patients with coronary graft vasculopathy. Usually, everolimus is initiated no earlier than six months after heart transplantation. Prednisolone 5 mg daily is commonly prescribed long-term. This approach covers most immunosuppressive protocols at our center. In individual cases with a high risk of rejection or reduced kidney function requiring dose adjustments of calcineurin inhibitors, a quadruple immunosuppression regimen is employed.

For lung transplant recipients: Standard induction involved basiliximab (except for cystic fibrosis and bronchiectasis cases) plus steroids. Maintenance usually includes a triple regimen of tacrolimus (initial target trough levels: 8–10 ng/mL, reduced after 12–24 months), mycophenolate mofetil (up to 2000 mg/day), and low-dose prednisolone (5 mg/day). In cases of tacrolimus intolerance, cyclosporine A (target levels: 150–200 ng/mL) was used. Everolimus is generally introduced at least three months after transplant for specific indications, such as recurrent CMV infections or malignancy.

Medical records of all patients were reviewed independently by two researchers (SP and LK). The study was conducted in accordance with the recommendations of the local ethics committee. Statistical analyses were conducted using SPSS software, version 29.01.0 (IBM SPSS Statistics, Chicago, IL, USA). A p-value less than 0.05 was considered statistically significant.

From 2012 to September 2018, an in-house PCR assay based on well-established primers and protocols was used for HEV detection (Forward: JVHEVF 5′-GGTGGTTTCTGGGGTGAC-3′; Reverse: JVHEVR 5′-AGGGGTTGGTTGGATGAA-3′; Probe: JVHEVP 5′-FAM-TGATTCTCAGCCCTTCGC-MGB-3′). Since October 2018, HEV PCR diagnostics have been performed using the fully Automated Roche cobas system (Roche, Mannheim, Germany). For detecting anti-HEV IgG and IgM antibodies, the Mikrogen recomblot (Mikrogen, Neuried, Germany) was used from 2012 until October 2021. Starting November 2021, the Roche CLIA has been employed to detect anti-HEV IgG and IgM antibodies (Roche, Mannheim, Germany).

The University Medical Center Hamburg-Eppendorf does not have specific recommendations for ribavirin dosage. If the hemoglobin level drops below 10 mg/dL, the dose is usually reduced, especially in patients with impaired renal function (GFR < 50 ml/min). At our center, ribavirin therapy is given for 3 months if viral negativity occurs within one month of starting treatment, and for 5 months if PCR negativity happens more than one month after therapy begins.

## Results

### Characteristics of total cohort

Detailed characteristics of the patient cohort and the various subgroups—liver transplant, kidney transplant, heart transplant, and lung transplant recipients—are shown in Table 1.

Between June 2011 and June 2025, a total of 68 solid organ transplant (SOT) recipients were diagnosed with hepatitis E virus (HEV) infection. Five patients were excluded from further analysis: one liver transplant recipient had previously undergone stem cell transplantation and therefore had a significant different level of immunosuppression compared to the rest of the liver transplant group. Four additional patients were lost to follow-up. These individuals were from other cities—or, in one case, another country—and no follow-up data were available after their initial HEV diagnosis.

A total of 63 SOT recipients (67% male; average age 53 years, range 20–83) with PCR-confirmed HEV infection were included in the analysis. Of these, 40 patients (63%) met the criteria for chronic HEV infection.

Excluding the liver and stem cell transplant recipients and the four patients lost to follow-up, 52 of 63 patients (83%) cleared the infection either spontaneously or after uncomplicated ribavirin therapy. This included 47 of 52 abdominal organ transplant recipients (90%) and 6 of 11 thoracic organ transplant recipients (55%) (*p* = 0.02).

Additionally, one heart transplant recipient achieved viral clearance after a second course of ribavirin, and 10 patients could not clear the infection despite extended ribavirin therapy. These patients were treated with low-dose maintenance ribavirin therapy (200 mg daily). Among them, one kidney transplant recipient cleared the infection while on maintenance therapy. Four patients died during treatment, and the remaining five are still receiving 200 mg of ribavirin daily at the time of analysis.

The proportion of patients with chronic infection was lower among recipients of abdominal organs (liver or kidney) at 58% (30/52) compared to thoracic organ recipients (heart or lung) at 91% (10/11). However, this difference was only borderline statistically significant (*p* = 0.047, Chi-square test). In contrast, the rate of sustained virological response (SVR) was significantly higher in abdominal organ recipients (90%, 47/52) than in thoracic organ recipients (55%, 6/11; *p* = 0.02). Neither viral load in blood or stool nor ALT levels differed significantly between abdominal and thoracic organ recipients (Mann–Whitney test).

### Characteristics of HEV infected transplant recipients in comparison to the total cohort of transplant recipients at our center

To compare the distribution of sex and transplanted organs in the HEV-infected group with that of the overall transplant population at the University Medical Center Hamburg-Eppendorf, all solid organ transplant recipients from the past 10 years were included as controls (*n* = 1,855). There were no significant differences between the HEV-infected group (*n* = 67) and the total transplant group (*n* = 1,855) in terms of sex distribution (67% male [45/67] vs. 60% male [1,109/1,855], p = not significant) or the distribution of transplanted organs: liver (43% [29/67] vs. 41% [752/1,855]), kidney (37% [25/67] vs. 43% [793/1,855]), heart (13% [9/67] vs. 11% [200/1,855]), and lung (6% [4/67] vs. 6% [110/1,855]) (all p = not significant).

### Evidence of extrahepatic manifestations of HEV in transplant recipients?

In four patients, evidence of extrahepatic manifestations could be observed (Table 1). Three patients suffered from arthralgia, and one patient (a 51-year-old heart transplant recipient) developed neuralgic amyotrophy, a well-known neurological manifestation of HEV infections. Interestingly, this episode occurred despite ongoing ribavirin therapy. This patient has been described previously in a publication [26]. At the time of his neurological symptoms, he had no detectable viral load in his blood but still had a detectable viral load in his ejaculate. Low HEV positivity (< 24 IU/ml) was detected in his cerebrospinal fluid.

### Serological status

A total of 59 patients were tested for anti-HEV IgG and IgM antibodies. Of these, 36 (61%) tested positive for both anti-HEV IgG and IgM. No patients tested positive for only IgG or only IgM. Among the 36 seropositive patients, 72% were male (26/36).

Regarding infection status, 67% of seropositive patients (*n* = 24) had a chronic HEV infection, 28% (*n* = 10) had an acute infection, and 6% (*n* = 2) were lost to follow-up. Among seropositive individuals, 72% (*n* = 26) achieved sustained virological response (SVR), 22% (*n* = 8) remained chronically infected, and 6% (*n* = 2) were lost to follow-up. Notably, all three deaths in the cohort occurred among seropositive patients.

The average age of seropositive patients was 54 years (range 20–82, SD 16). The average viral load was 15,825,860 IU/ml (range 72–240,000,000 IU/ml, SD 45,879,188), and the average ALT level was 351 U/ml (range 15–3,032 U/ml, SD 531).

There were no significant differences between seropositive and seronegative patients in terms of sex distribution or infection chronicity (Chi-square test, p = not significant). Surprisingly, seropositive patients achieved SVR significantly less often than seronegative patients (26/36 vs. 22/22, *p* < 0.001). No significant differences were observed between the groups regarding age (*p* = 0.09), viral load (*p* = 0.39), or ALT levels (*p* = 0.14).

### Ribavirin treatment

Ribavirin therapy was initiated in 43 patients (64%). Of these, 40 had chronic HEV infection, while in three patients, ribavirin was started before their viremia lasted more than three months, based on their doctors’ decisions. Consequently, these three patients did not meet the criteria for a chronic infection and were excluded from further analysis related to ribavirin treatment.

Of the 40 patients with chronic infection who received ribavirin, 31 (78%) cleared the virus, while HEV persisted in nine patients (22%). Patient details for the chronically infected individuals treated with ribavirin are listed in Table 2. All nine patients who did not achieve viral clearance despite re-treatment and/or extended low-dose ribavirin therapy (200 mg daily) remained clinically stable, and none died from direct complications of cirrhosis. However, two of the patients who died had cirrhosis, and the connection between their deaths and cirrhosis remains uncertain; cirrhosis may have contributed to worsening their conditions.

Interestingly, among patients with chronic infection who achieved sustained virological response (SVR) without requiring maintenance ribavirin therapy (*n* = 30), 52% tested positive for both anti-HEV IgG and IgM at diagnosis. Conversely, among patients who did not clear the infection despite 6 months of ribavirin treatment (*n* = 10), all 9 available serum samples tested positive for both anti-HEV IgG and IgM (*p* = 0.01, Table 2).

In 22 out of 40 chronically HEV-infected transplant recipients (55%), the ribavirin dose was reduced. Specifically, this included 4 out of 13 liver transplant patients (31%), 11 out of 17 kidney transplant patients (65%), 5 out of 7 heart transplant patients (71%), and 2 out of 3 lung transplant patients (67%). Consequently, ribavirin dose reduction was needed in 15 of 30 abdominal organ recipients (50%) and in 7 of 10 thoracic organ recipients (70%) (p = ns).

### Role of immunosuppression in HEV infection progression

In the cohort of analyzed transplant recipients (*n* = 63), 8 received mono-immunosuppression (13%), 30 dual immunosuppression (47%), and 25 triple immunosuppression (39%). Forty-one patients received tacrolimus (62%), 12 cyclosporine A (19%), 34 mycophenolate mofetil (or enteric-coated mycophenolate mofetil) (53%), 25 everolimus (39%), 3 azathioprine (5%), and 30 received steroids (47%). In 13 patients (20%), immunosuppression was reduced after HEV infection was detected; 12 of these developed chronic HEV infection, while 1 cleared the infection spontaneously without ribavirin. However, an analysis of the relationship between final HEV clearance and reduction of immunosuppression showed that 8 out of 12 (67%) patients with reduced immunosuppression ultimately cleared the virus, compared to 36 out of 41 (86%) in the group without reduction (*p* = 0.027). When analyzing only the subgroup of 40 patients who received ribavirin, it appears that 6 out of 11 (55%) in the reduced immunosuppression group cleared the infection, whereas 25 out of 29 (86%) in the group without immunosuppression reduction did so (*p* = 0.047). Reduction of immunosuppression was observed in 5 out of 13 liver transplant recipients (38%), 2 out of 17 kidney recipients (12%), 2 out of 7 heart transplant recipients (29%), and 2 out of 3 lung transplant recipients (67%) among the 40 patients with chronic HEV infection.

Neither the use of tacrolimus, cyclosporine A, mycophenolate mofetil, azathioprine, nor steroids was associated with a significantly increased likelihood of developing chronic infection or achieving final clearance. However, 4/25 (16%) of patients receiving everolimus cleared the infection spontaneously, compared to 20/39 (51%) of patients on regimens without everolimus (*p* = 0.007). Despite this, there was no significant association between everolimus use and final clearance of infection. Additionally, the SVR rate at the end of our observational study did not differ significantly (*p* = 0.137) between everolimus-treated patients (19/25; 76%) and those not treated with everolimus (36/39; 92%).

### Deceased patients

Four patients (6%) died while still HEV viremic. All had chronic hepatitis E and did not clear the virus after five months of ribavirin therapy. Even with subsequent low-dose maintenance therapy of 200 mg ribavirin daily, the infection persisted in these patients.

The first deceased patient was a 58-year-old male kidney transplant recipient with pneumonia and sepsis. He had undergone transplantation due to polycystic organ degeneration, and his entire liver was cystic, with no signs of cirrhosis.

The second deceased patient was also a kidney transplant recipient who had liver cirrhosis (FibroScan 45 kPa, esophageal varices, and a history of ascites decompensation). The 55-year-old man had no identifiable cause for the cirrhosis other than chronic hepatitis E. He developed a severe COVID-19 infection followed by pneumonia and sepsis, which ultimately led to his death.

The third patient was a 58-year-old heart transplant recipient with cirrhosis caused solely by chronic HEV infection. Retrospective testing of stored serum samples showed that he had received plasma transfusions along with rituximab during an acute rejection episode about one year before being diagnosed with HEV infection (almost three years after his transplant). These plasma transfusions were given through plasmapheresis before routine HEV screening of blood products began in Germany in January 2020. Unfortunately, he developed bronchial carcinoma, which recurred after surgery, and he died from the cancer. The fourth patient, who had died, was stabilized on ribavirin maintenance therapy for her chronic hepatitis E but died due to prolonged failure of her lung transplant.

## Discussion

### Putting our findings in context

This large, single-center observational cohort study examined 63 solid-organ transplant (SOT) recipients with HEV infection, including liver, kidney, heart, and lung transplant recipients at a major transplant center in northern Germany. The distribution of organ types among HEV-infected patients did not differ significantly from the overall distribution of transplanted organs at the University Medical Center Hamburg-Eppendorf over the past 10 years. Therefore, there is no increased risk of HEV exposure among recipients of different organs.

However, recipients of thoracic organs developed chronic HEV infection more frequently than recipients of abdominal organs, with borderline statistical significance (*p* < 0.05). This finding may be due to the generally higher level of immunosuppression in heart and lung transplant recipients compared to liver or kidney transplant patients. Alternatively, differences in HEV testing practices across departments may have led to variations in the timing of diagnosis.

More striking, however, was the difference in treatment outcomes: 91% of liver or kidney transplant recipients cleared the virus, compared to only 55% of heart or lung transplant recipients (*p* = 0.007). These findings suggest that thoracic organ recipients are a high-risk group for HEV treatment failure. This observation is interesting, considering that the ribavirin dose had to be reduced in 70% of thoracic organ recipients but in only 50% of abdominal organ recipients, and that a reduction in immunosuppressive dose was attempted in 23% of abdominal organ recipients but in 40% of thoracic organ recipients. The benefit of reducing the immunosuppressant dose should therefore not be overestimated. This finding aligns with previous reports demonstrating that reducing immunosuppression without ribavirin results in clearance in only about 15% of chronically infected patients [27].

This observation has important clinical implications. Current EASL guidelines for treating chronic HEV infection are mainly based on data from liver and kidney transplant recipients [25]. However, our study shows that heart and lung transplant recipients are a particularly challenging group to treat. While the EASL guidelines recommend a six-month treatment duration for transplant recipients with chronic hepatitis E, our findings suggest that specific guidance may be needed for recipients of different organs. Future multicenter studies should investigate whether recipients of thoracic organs should have longer treatment than those with abdominal organs. A key challenge for future treatment strategies is to balance personalized therapies—considering individual factors and ribavirin serum levels—with broader recommendations.

Furthermore, multicenter studies or registries are needed to determine the optimal dosage and treatment duration for recipients of different organs. These studies should also explore the clinical significance of specific HEV genome mutations—particularly A1343V and G1634R—that have been associated with reduced ribavirin effectiveness. Unfortunately, mutation testing was unavailable at our center and was not performed in this study.

### Risk factors for HEV exposure

It is also noteworthy that although HEV-infected transplant recipients in our cohort were more often male than female, this simply reflects the overall sex distribution among all transplant recipients at our center over the past 10 years (67% male [45/67] in the HEV cohort vs. 60% male [1,109/1,855] in the total cohort; p = not significant). Therefore, the well-documented higher consumption of raw or undercooked pork among men compared to women does not appear to be linked to an increased risk of HEV infection in male transplant recipients. This suggests that other sources of HEV exposure could also be relevant for this population. Recently, swine-derived pancreatic enzymes have been identified as a potential HEV transmission [28, 29]. However, other yet unidentified sources may also contribute and warrant further investigation.

### Value of HEV specific antibodies

Another important finding of this study is that 34% of solid organ transplant recipients tested seronegative (anti-HEV IgG/IgM negative) despite having PCR-confirmed HEV infection. This supports previous recommendations that serological testing alone is unreliable in immunosuppressed patients and that PCR should be the diagnostic standard in this population, as outlined in the EASL guidelines.

Unexpectedly, patients who tested positive for anti-HEV IgG and IgM at diagnosis were significantly less likely to clear the virus compared to seronegative patients (see Table 2). The reason for this remains unclear. Although speculative at this point, this observation raises questions about the potential role—or limitations—of HEV-specific immunoglobulins in transplant recipients.

### Ribavirin therapy and long-term management of chronically HEV infected patients

**Fig. 1:**
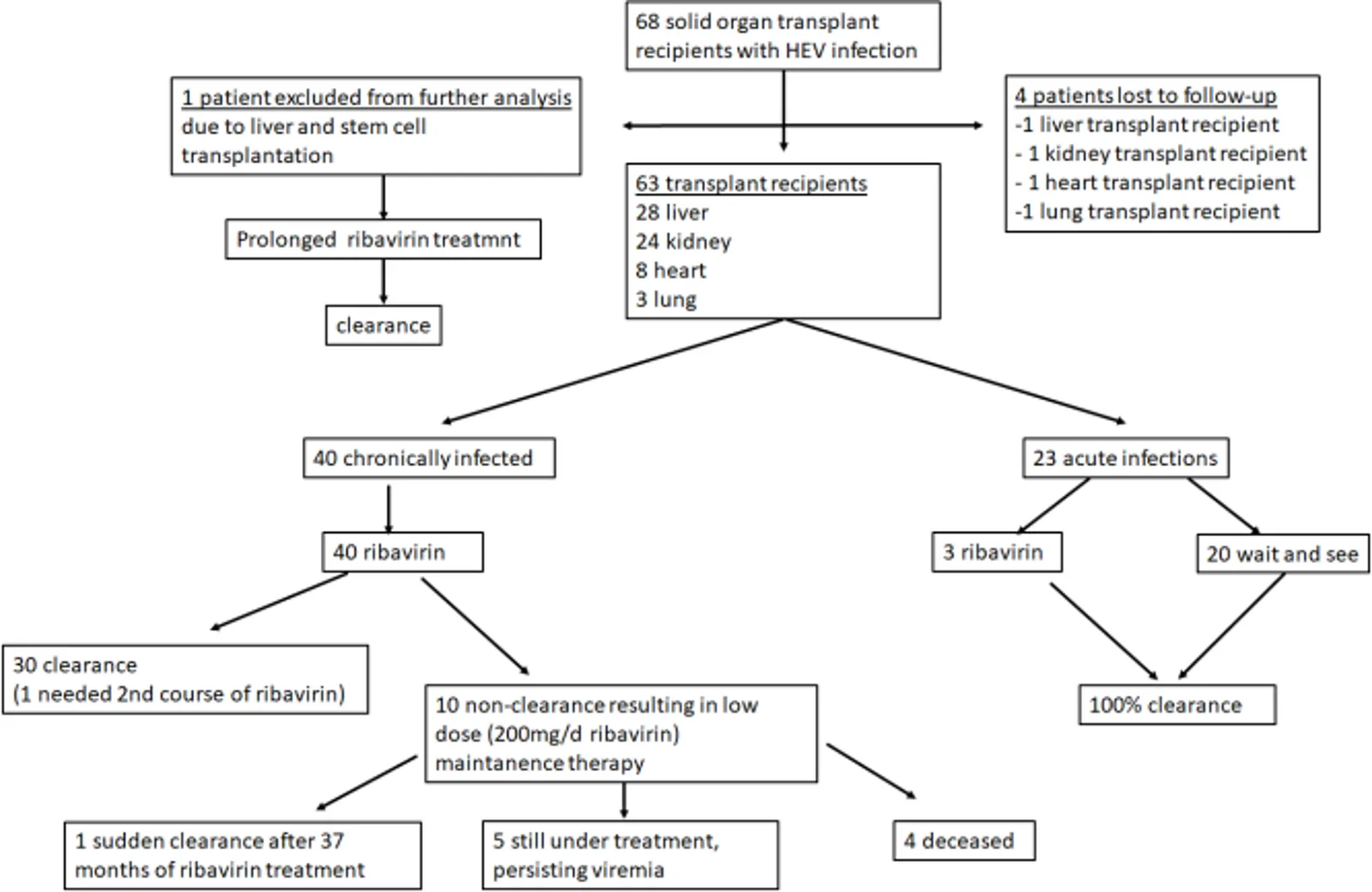
Outcome of acute or chronic HEV infection

In our cohort, 78% of solid organ transplant recipients with chronic HEV infection who received ribavirin achieved viral clearance (see Fig. 1; Table 2). This result aligns with previously published data, despite the higher proportion of thoracic organ recipients in our cohort, who are generally more immunosuppressed and often underrepresented in earlier studies. Additionally, our experience indicates that long-term, low-dose maintenance therapy with ribavirin (200 mg/day) can stabilize liver function in most patients who do not initially respond to standard treatment. In nearly all cases, liver function tests normalized during maintenance therapy, except for one patient. In this heart transplant recipient, comorbid diabetes, hepatic steatosis, and cirrhosis strongly suggest additional contributing factors. Notably, one patient achieved viral clearance after several years of maintenance therapy, highlighting that even in cases of apparent treatment failure, long-term viral suppression and eventual cure remain achievable goals.

### Mortality and clinical implications

Of the nine patients who remained chronically infected despite treatment, four died (44%). However, because of the retrospective nature of this study, a direct causal link between persistent HEV infection and death cannot be confirmed. The causes of death included sepsis and pneumonia (*n* = 2), graft failure (*n* = 1), and metastatic bronchial carcinoma (*n* = 1). Notably, three of these four patients had liver cirrhosis, which could only be attributed to chronic HEV infection. Whether these patients would have survived without chronic HEV (and cirrhosis) remains speculative. Although this study shows that thoracic organ recipients tend to have a more difficult course of hepatitis E compared to abdominal organ recipients, these results are based on a single-center cohort. Larger multicenter studies are necessary.

## Conclusions

Based on our retrospective cohort study, the key conclusions that can be drawn may have lasting implications for the clinical management of solid organ transplant recipients with HEV infection.

Thoracic organ transplant recipients are a high-risk group for chronic HEV infection and treatment failure. This seems to be because of the stronger immunosuppressive drugs used in these patients compared to those with abdominal organ transplants. This emphasizes the need for more aggressive treatment strategies in this subgroup—either through extended ribavirin therapy or by using new antiviral agents.

The impact of everolimus-containing immunosuppression on the course of an HEV infection should not be overestimated. Although patients receiving everolimus developed chronic hepatitis E more frequently than others in our study, these were mostly patients undergoing triple immunosuppression; for mycophenolate, the alternative component of triple immunosuppression, a possible antiviral effect on HEV replication is assumed.

PCR-based diagnostics should be regarded as the gold standard for detecting HEV infection in immunosuppressed transplant recipients. Serological testing has proven unreliable and should not be used as the only diagnostic method in this patient group.

The benefits of tacrolimus-free therapy and reduced immunosuppression mentioned earlier should not be overstated.

Low-dose maintenance therapy with ribavirin (200 mg/day) is a feasible option for long-term viral control in patients who do not achieve sustained virological response with standard therapy. While viral clearance is uncommon, biochemical stabilization can be reached in most patients, and in some cases, even complete viral eradication may happen over time.

Individualized treatment plans that consider all of the patient’s clinical parameters are preferable to strict, one-size-fits-all recommendations regarding dose reduction of immunosuppressants and ribavirin.

**Table 1:** Patients characteristics (patients with unknow outcome censored)

	Total cohort (*n* = 63)	Liver transplant (*n* = 28)	Kidney transplant (*n* = 24)	Heart transplant (*n* = 8)	Lung transplant (*n* = 3)
Male	44 (70%)	17 (61%)	17 (71%)	7 (78%)	2 (67%)
Chronic infection Acute infection	41 (65%) 22	13 (31%) 14	17 (71%) 7	7 (78%) 1	3 (100%) 0
Anti HEV IgG*					
Positive Negative Missing	37 21 7	8 13 5	14 9 1	7 0 1	3 0 0
Anti HEV IgM*					
Positive Negative Missing	36 23 8	6 15 5	17 6 1	7 0 1	2 1 0
Extrahepatic manifestations	4 (6%) -1 Neuralgic amyotrophy -3 Arthralgia	1 (4%) -1 Arthralgia	0 (0%)	3 (38%) − 1 Neuralgic amyotrophy − 2 Arthralgia	0 (0%)
Diabetes	18 (27%)	5 (19%)	7 (29%)	4 (50%)	0 (0%)
Hypertension	44 (66%)	15 (56%)	18 (72%)	5 (63%)	2 (67%)
Coronary heart dis.	8 (12%)	1 (4%)	5 (16%)	1 (11%)	1 (33%)
Hyperlipidemia	18 (27%)	6 (22%)	3 (12%)	5 (63%)	2 (67%)
Death	4 (6%)	0 (0%)	2 (8%)	1 (13%)	1 (33%)
SVR					
Clearance No clearance	53 (84%) 10 (16%)	28 (100%) 0 (0%)	19 (79%) 5 (21%)	4 (50%) 4 (50%)	2 (67%) 1 (33%)
Age, mean (stdw)	53 (14.4)	51 (15.9)	52 (12.2)	55 (13.9)	64 (12.0)
Viral load blood U/ml, mean* (*n* = 67/67 positive) Viral load stool, U/ml, mean* (*n* = 29/30 positive)	13.387.188 19.899.388	17.524.699 31.266.925	4.859.122 3.240.248	28.730.148 2.790.250	7.416.325 27.343.365
ALT, U/l*	378 (481.3)	345 (369.4)	469 (657.7)	218 (180.5)	190 (177.1)

*At diagnosis

**Table 2:** Comparison of chronically infected, ribavirin treated patients with or without SVR

	Clearance within 6 months or less riba treatment 30/40	No clearance within max 6 months riba treatment 10/40	*p*-value
Sex	M = 19, F = 11	M = 9, F = 1	ns
Ribavirin dosage; mg ribavirin/ kg body weight, mean (range)	8.4 (2,7–13,3)	6,3 (1,3–13,3)	ns
Ribavirin reduction necessary	14 (47%)	8 (80%)	ns
Reduction of immunsosuppression	6 (20%)	5 (50%)	ns
Tacrolimus	18 (60%)	7 (70%)	ns
Cislosporine	6 (20%)	0 (0%)	ns
Mycophenolate mofetil	14 (47%)	6 (60%)	ns
Everolimus	15 (50%)	6 (60%)	ns
Azathioprine	3 (10%)	0 (o%)	ns
Steroids	13 (43%)	7 (70)	ns
Age, mean (range) in years	52 (20–79)	55 (36–71)	ns
Viral load blood at diagnosis, mean (range) in IU/ml	13.388.138 (3.700–210.000.000)	4.048.972 (min = 100.000-20.577.850)	ns
Viral load stool at diagnosis, mean (range) in IU/ml (available for 22 patients)	33.463.610 (700 − 130.000.000)	5.175.117 (180.000–15.000.000)	ns
ALT at diagnosis, mean (range) in U/l	295 (15-1463)	164 (28–516)	ns
Creatinine at diagnosis, mean (range) in mg/dl	1.7 (0.6–5.8)	1.8 (0.8–2.5)	ns
Creatinine during ribavirin treatment, mean (range) in mg/dl	1.7 (0.8–5.9)	2.5 (1.8–3.5)	ns
Creatinine after ribavirin treatment, mean (range, in mg/dl	2.2 (0.8–8.71)	3.27 (in the maintanence cohort only one patient cleared the infection)	ns
Time since transplantation in months	93 (5-288)	91 (12–228)	ns
Death	0	4	0.004
Anti HEV IgG at diagnosis positive/negative (1 missing)	15 (52%)	9/0	0.01
Anti HEV IgM at diagnosis positive/negative (1 missing)	15 (52%)	9 (100%)	0.01
Immunosuppression (at diagnosis):			
Tacrolimus Ciclosporin MMF Everolimus Azathioprine Prednisolone	17 6 15 16 2 12	7 0 6 6 0 7	ns ns ns ns ns ns

## Data Availability

No datasets were generated or analysed during the current study.
